# Clarification of Misleading Perceptions of COVID-19 Fatality and Testing Rates in Italy: Data Analysis

**DOI:** 10.2196/19825

**Published:** 2020-06-17

**Authors:** Davide Tosi, Alessandro Verde, Manuela Verde

**Affiliations:** 1 Department of Theoretical and Applied Science University of Insubria Varese Italy; 2 Heart Failure Unit, Niguarda Cardio Center Cardiothoracic and Vascular Department ASST Great Metropolitan Hospital Niguarda Milan Italy; 3 CRIMEDIM Research Centre in Emergency and Disaster Medicine Translational Medicine Department Università del Piemonte Orientale Novara Italy

**Keywords:** COVID-19, SARS-CoV-2, fatality rate, swab tests, Italy, Lombardy region

## Abstract

**Background:**

The fatality rate of coronavirus disease (COVID-19) in Italy is controversial and is greatly affecting discussion on the impact of containment measures that are straining the world’s social and economic fabric, such as instigating large-scale isolation and quarantine, closing borders, imposing limits on public gatherings, and implementing nationwide lockdowns.

**Objective:**

The scientific community, citizens, politicians, and mass media are expressing concerns regarding data suggesting that the number of COVID-19–related deaths in Italy is significantly higher than in the rest of the world. Moreover, Italian citizens have misleading perceptions related to the number of swab tests that have actually been performed. Citizens and mass media are denouncing the coverage of COVID-19 swab testing in Italy, claiming that it is not in line with that in other countries worldwide.

**Methods:**

In this paper, we attempt to clarify the aspects of COVID-19 fatalities and testing in Italy by performing a set of statistical analyses that highlight the actual numbers in Italy and compare them with official worldwide data.

**Results:**

The analysis clearly shows that the Italian COVID-19 fatality and mortality rates are in line with the official world scenario, as are the numbers of COVID-19 tests performed in Italy and in the Lombardy region.

**Conclusions:**

This up-to-date analysis may elucidate the evolution of the COVID-19 pandemic in Italy.

## Introduction

Since February 2020, the severe acute respiratory syndrom coronavirus 2 (SARS-CoV-2) pandemic has generated much concern among citizens and mass media in Italy regarding the real and official numbers provided by the Italian Government and the Italian Department of Civil Protection [1-4]. One of the most discussed topics is why the case fatality rate in Italy appears to be much higher than in other countries. Another main topic of debate is why inappropriate numbers of COVID-19 tests appear to have been performed on citizens of Italy in general and the Lombardy region in particular, thus underrepresenting the infected population.

The purpose of this study is to use a set of statistical and data analyses to clarify both the actual and official case fatality rate in Italy in comparison to those in other countries as well as the actual size of the tested population.

## Methods

We examined data regarding coronavirus disease (COVID-19) case fatality rates and numbers of throat swab and nasal swab tests using real time polymerase–chain reaction assay methods conducted in world populations to describe the actual global picture of the case fatality rate of COVID-19 in Italy compared to other industrialized countries and the number of tests performed since the first outbreak in Italy (Codogno, Lodi province, and the Lombardy region) between February 24 and April 14, 2020. This time interval was selected to cover most of the COVID-19 epidemic curve. Raw data for the case fatality rate and swab tests were obtained as comma-separated value (.csv) open files from the Italian Department of Civil Protection [1] and from the Worldometer portal [5]. Data regarding the critical situation in the Lombardy region were obtained as open data directly from [1]. It is important to highlight that at the time of writing, the March 22, 2020 decree (Implementation of the decree 23 February 2020 No. 6: Urgent measures for the containment and management of the COVID-19 epidemiological emergency (20A01807), Official Gazette: General Series No.76 of 22-03-2020 [6]) is in action to temporarily stop all unnecessary industrial and commercial production activities. Currently, all of Italy is in a quarantine red zone.

To compare the situations in Italy and in other countries, the analysis was limited to the Group of Ten (G10) industrialized countries: Belgium, Canada, France, Germany, Japan, Italy, the Netherlands, the United Kingdom, the United States, and Sweden. For the Lombardy region analysis, the most affected regions and areas in each G10 country were identified and compared. The case fatality rate trend in Italy was plotted using Excel software (Microsoft Corporation), while all the other statistical analyses (absolute values, mean values, and normalized values) are reported in tables.

## Results

### COVID-19 Fatality Rate in Italy

To clarify both actual and official case fatality rates in comparison to those of other countries as well as the actual extent of the tested population, it is important to differentiate the case fatality rate from the actual fatality rate (infection fatality rate). The case fatality rate is the ratio of the number of deaths to the total number of positive tests, while the infection fatality rate is the ratio of the number of deaths to the total infected population. However, because the entire population (symptomatic and asymptomatic) cannot be tested with sufficient speed, it is necessary to rely on case fatality rates for epidemiology measures and policy planning. Moreover, as the epidemic escalates, the tested population represents the emergent cluster of patients who, by seeking care, turn to and contact national health systems.

At present, the COVID-19 case fatality rate in Italy is 12.80% [5]. In China, the case fatality rate shows a nonlinear trend, with a high rate at the start of the outbreak (17.3%) that decreased to 0.7% at the end of the emergency. China hit the infection curve plateau on February 20, 2020. At the time of writing, the Chinese case fatality rate is approximately 4.1% [5].

The current Italian case fatality rate is comparable with that of other European countries, such as Belgium (12.80%), the United Kingdom (12.70%), France (11.20%), and Spain (10.44%). To date, the worldwide case fatality rate is 6.40% [5]. In Germany, the case fatality rate is 2.7%; however, the Robert Koch Institute has stated that this rate is expected to grow greatly [7]. Furthermore, general misalignment with other European countries is observed for other German indices related to COVID-19, such as the distribution of deaths per age of infected people and the distribution of deaths per gender [8,9].

The Lombardy region is the region most affected by COVID-19 worldwide; data indicate that this region represents 38% of the total infections in Italy, with a regional case fatality rate of 18.08% [1]. No other countries have such unbalanced distribution and variability among their regions [5]; therefore, we can perform the statistical exercise of considering the Lombardy region value as an outlier (ie, a data point that differs significantly from the other regional observations). Hence, if we attempted to disaggregate the contribution of the Lombardy region proportionally to the national datum, we would obtain an Italian case fatality rate of 9.60%. The Italian case fatality rate value would decrease even further to 7.9% if data from the Emilia-Romagna region (the second most affected Italian region) were also excluded.

This statistical exercise stems from the consideration that the very first COVID-19 outbreak in Europe occurred in the Lombardy region, and the National Health System was realistically unprepared to face a pandemic.

**Figure 1:**
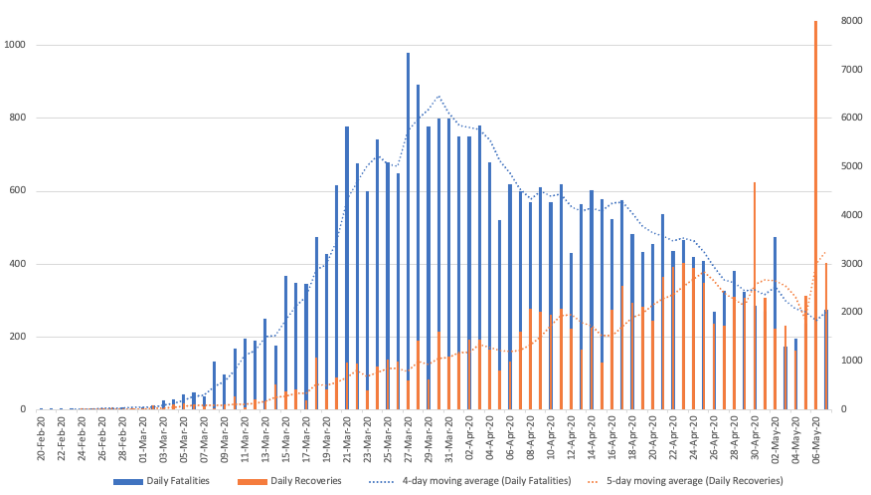
Graphical plot of COVID-19 daily fatalities versus recoveries in Italy. COVID-19: coronavirus disease.

Ultimately, it is important to note that the case fatality rate trend is not linear over time due to the number of total infected people depending on the daily number of new infections combined with the numbers of recovered and discharged patients. As shown in Figure 1, the trend line (ie, the 5-day moving average) of daily recoveries grows strongly over time, whereas the trend line for COVID-19 fatalities decreases with a different slope function.

**Figure 2:**
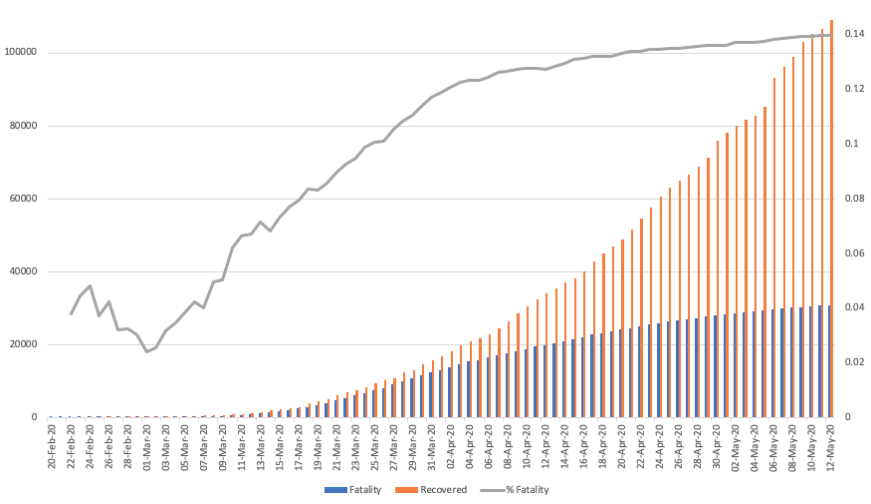
Graphical plot of COVID-19 cumulative total fatalities versus recoveries in Italy. COVID-19: coronavirus disease.

Figure 2 also shows that the curve of total recovered/discharged patients grows much faster than the curve of total deaths. The difference in the slopes of the two curves could enable a reliable prediction of a decrease in the national case fatality rate at the end of the first wave of the epidemic.

Furthermore, as officially reported in the available open data [1], the occupancy of beds in COVID-19–dedicated intensive care units is steadily decreasing, with increasingly diverging curves between the number of new infections and the number of patients with severe symptoms that could lead to death in the near future. At the time of writing, there are more than 3000 patients with severe symptoms out of 104,000 total active positive cases.

Table 1 shows the COVID-19 case fatality rates and mortality rates (the ratio of deaths of patients who tested positive for COVID-19 to the total number of citizens) on April 14, 2020 among the industrialized countries in the G10: Belgium, Canada, France, Germany, Japan, Italy, the Netherlands, the United Kingdom, the United States, and Sweden [5]. Comparing the Italian data to those of the other G10 countries, the COVID-19 mortality in Italy is approximately 358 per 1 million inhabitants, which is not the highest value in the set. Belgium, for instance, has a mortality rate of 419 per 1 million inhabitants, and the rate in Spain is 409 per 1 million inhabitants. Following the model of the statistical exercise above, if we excluded the Lombardy region mortality rate of 1000 per 1 million inhabitants, the Italian mortality rate would decrease to 220 per 1 million inhabitants.

**Table 1:** Comparison of the case fatality rate and mortality rate in Italy and the Lombardy region with those of the other G10 countries.

Country	Case fatality rate, %	Mortality rate, deaths per 1 million inhabitants
Belgium	12.80	419
Canada	4.00	32
France	11.20	263
Germany	2.70	46
Italy	12.80	358
Japan	2.12	2
Lombardy region	18.08	1,000
Netherlands	11.40	193
United Kingdom	12.70	190
United States	5.50	86
Sweden	10.50	132
Spain	10.44	409

### COVID-19 Test Coverage in Italy

One of the most debated issues in Italy is the number of COVID-19 tests performed [1]; the scientific community is divided by suggestions of a hypothetical high number of undiagnosed cases that would lower the national case fatality rate. This aspect can be easily assessed from the officially declared world data [5]. As reported in Table 2, at the time of writing (April 15, 2020), approximately 1,100,000 COVID-19 tests had been performed in Italy. In the United States, where over 600,000 COVID-19 cases had been confirmed to date, 3,300,000 tests were performed. In Spain (the second most affected country in the world, with over 170,000 total cases), only 600,000 tests were performed; 330,000 tests were performed in France, 1,300,000 in Germany, and 380,000 in the United Kingdom. Therefore, in absolute terms, the nation of Italy has performed the third largest number of tests in the world.

After weighting the number of tests by the population of each nation, Italy has a ratio of 17,800 tests per 1 million inhabitants, ranking first among the G10 countries in terms of tests per 1 million inhabitants (column 3 of Table 2) [5]. This gap is even more evident for the Lombardy region, which has a ratio of 25,000 tests per 1 million inhabitants.

Conversely, if we consider the total of patients who tested positive and the number of swabs performed in the equation, we find that the lower the percentage ratio, the more effectively the nation has tested its citizens.

It is important to observe the temporal misalignment between all the countries because the COVID-19 outbreak started in different countries on different dates. Hence, these values should be normalized by the cumulative number of days since the first outbreak in each country. However, it should be noted that the Lombardy region suddenly discovered the first outbreak with a delay of at least 18 days (ie, 2 weeks of average SARS-CoV-2 incubation time plus 4 or 5 days to develop severe symptoms). In contrast, other European Union countries were able to prepare in advance and may have benefited from a significant advantage in managing the diffusion of COVID-19 by observing the behavior of the COVID-19 spread in Italy, particularly in the Lombardy region.

**Table 2:** COVID-19 testing data for Italy and the Lombardy region and for the other G10 countries.

Country	Total tests	Tests per 1 million inhabitants	Positive cases/tests, %
Belgium	134,000	11,000	26.0
Canada	450,000	12,000	6.7
France	330,000	5,000	44.3
Germany	1,300,000	16,900	8.0
Japan	100,000	800	9.0
Italy	1,100,000^a^	17,800^b^	14.8^c^
Netherlands	140,000	8,500	20.0
United Kingdom	380,000	6,000	24.6
United States	3,300,000	10,000	19.8
Sweden	70,000	7,000	16.6
Lombardy region	230,000	23,000	27.9
Spain	600,000	17,500	19.7

^a^Third place.

^b^First place.

^c^Fourth place.

### Additional Considerations in the Lombardy Region

As already discussed, the Lombardy region has a very significant impact on the national data in Italy. Hence, it may be worthwhile to explore additional considerations to better understand the impact of the Lombardy region on the global data. The Lombardy region has 10,000,000 inhabitants in a territory of 23,800 square kilometers and has a population density of 425 inhabitants/km^2^. Its capital city, Milan, is one of the most dynamic alpha cities in the world, with a population of 1,350,000 people and a population density that reaches 7600 units/km^2^. The Lombardy region also contains the largest logistics hubs in Italy (close to the first outbreak in Codogno [5]) and some of the largest in Europe. It is also one of the regions in Europe with the greatest flow of people and goods by air, rail, or road [10].

In this context, the analysis can be further extended by comparing the Lombardy region data with the most important regions/areas of the other G10 countries (see Table 3). If we focus on the total cases reported, the Lombardy region is the region with the second highest absolute value of COVID-19–positive cases (Lombardy region=59,000; New York City=123,000). If we normalize this value by the total number of inhabitants, New York, Antwerp, and Madrid have the highest numbers of positive cases per 1 million inhabitants. Regarding case fatality rates, the Lombardy region is the region with the highest number of fatalities (18.08%), followed by Île-de-France (17.99%) and Antwerp (14.20%). When we normalize the regional case fatality rate with the national datum (ie, the ratio of the regional case fatality rate to the national case fatality rate), New York City appears to have the highest multiplication factor (9.50%/5.50%) of ×1.73.

**Table 3:** COVID-19 statistics for the Lombardy region and for the most affected regions in the other G10 countries.

Country	Region	Total cases	Cases per 1 million inhabitants	Case fatality rate, %	Ratio of regional case fatality rate to national case fatality rate
France	Île-de-France	23,500	2000	17.00	1.52
Germany	Bayern	36,000	2800	3.20	1.18
Italy	Lombardy region	59,000	5900	18.08	1.41
Belgium	Antwerp	4500	9500	14.20	1.25
United Kingdom	London	19,500	2200	—^a^	—
Sweden	Stockholm County	7100	7500	14.10	1.34
Spain	Community of Madrid	48,000	8000	14.00	1.34
United States	New York City	123,000	13,700	9.50	1.73

^a^Not available.

## Discussion

The case fatality rate and mortality rate of COVID-19 in Italy are in line with the official world scenario. It is also evident that the numbers of COVID-19 tests performed in Italy and in the Lombardy region are comparable with those in the rest of Europe and the rest of the world.

## Abbreviations

COVID-19: coronavirus disease
csv: comma-separated value
G10: Group of Ten
SARS-CoV-2: severe acute respiratory syndrome coronavirus 2
